# Inflammatory based psychotic symptoms: when psychosis means encephalitis

**DOI:** 10.1192/j.eurpsy.2024.79

**Published:** 2024-08-27

**Authors:** M. Rojnic Kuzman

**Affiliations:** Zagreb University Hospital Centre and the Zagreb School of Medicine, Zagreb, Croatia

## Abstract

**Abstract:**

Schizophrenia, as one of the most common disorders from the psychotic spectrum is most commonly detected in the phase of first psychosis and may pose a diagnostic challenge, as commonly comprise a heterogeneous group of schizophrenias, with distinct clinical presentations. If it detected in its prodromal phase without clearly developed psychotic symptoms, the diagnosis is even more unreliable, as the transition to full blown psychosis in the next two years happens in 15-40% of more, depending probably on a variety of cumulative environmental risk factors (including childhood trauma, the use of high-potency cannabis, urbanicity, season of birth). Moreover, the first episode psychosis may underlie for example the first manic episode, brief intermittent psychotic symptoms in persons with borderline personality disorders, acute reaction to trauma, the use of cannabis and psychostimulants and different organics causes, such as endocrinologic disorders and autoimmune encephalitis. Therefore, in everyday clinical practice, the diagnosis of first episode psychosis always requires an assessment of possible causes of psychosis, and also factors that may influence prognosis and treatment. Usual assessment include detailed anamnestic and heteroanamnestic data, physical examination, standard blood laboratory findings, drugs in urine/ blood, EEG and CT/MR scan. The absence of typical risk factors for schizophrenia, as well as the absence of premorbid symptoms and developmental course typical for schizophrenia, abrupt course of psychotic symptoms, symptoms such as disorientation, catatonia, speech disturbances, alteration of consciousness, neurologic signs, autonomic dysfunction and laboratory aberrations may be especially indicative for organic cause and possibly encephalitis and require further confirmation with the analysis of cerebrospinal liquor with antineuronal antibodies.

**Disclosure of Interest:**

None Declared

